# Composition of low-molecular-weight glutenin subunits in common wheat (*Triticum aestivum* L.) and their effects on the rheological properties of dough

**DOI:** 10.1515/biol-2021-0059

**Published:** 2021-06-24

**Authors:** Sławomir Franaszek, Bolesław Salmanowicz

**Affiliations:** Institute of Plant Genetics, Polish Academy of Sciences, Strzeszyńska 34, 60-479 Poznań, Poland

**Keywords:** low-molecular-weight glutenin subunits, dough rheology, wheat, capillary zone electrophoresis, reverse-phase high-performance liquid chromatography

## Abstract

The main purpose of this research was the identification and characterization of low-molecular-weight glutenin subunit (LMW-GS) composition in common wheat and the determination of the effect of these proteins on the rheological properties of dough. The use of capillary zone electrophoresis and reverse-phase high-performance liquid chromatography has made it possible to identify four alleles in the *Glu-A3* and *Glu-D3* loci and seven alleles in the *Glu-B3* locus, encoding LMW-GSs in 70 varieties and breeding lines of wheat tested. To determine the technological quality of dough, analyses were performed at the microscale using a TA.XT Plus Texture Analyzer. Wheat varieties containing the *Glu-3* loci scheme (*Glu-A3b*, *Glu-A3f* at the *Glu-A3* locus; *Glu-B3a*, *Glu-B3b*, *Glu-B3d*, *Glu-B3h* at the *Glu-B3* locus; *Glu-D3a*, *Glu-D3c* at the *Glu-D3* locus) determined the most beneficial quality parameters.

## 1 Introduction

Wheat (*Triticum aestivum* L.) is a cereal species belonging to the family of Poaceae. Owing to its very good nutritional values (a rich source of starch, proteins, vitamins, minerals) and technological properties essential in the food industry, wheat is of great economic importance and is one of the most commonly grown cereals worldwide [1]. Analyses of the qualitative–quantitative composition of wheat storage proteins are a rich source of information regarding the technological properties of flour and are also used to select varieties in terms of desirable traits. The gluten complex, consisting of gliadins and glutenins, plays an important role during plant development and determines the technological use of wheat [2]. Glutenins are polymeric proteins that are divided into high-molecular-weight (HMW) glutenins with a molecular mass of 75–120 kDa and low-molecular-weight (LMW) glutenins with a mass of 20–55 kDa [3]. HMW glutenin subunits (HMW-GSs) account for nearly 10% of gluten proteins and determine 50–70% of the technological quality of the wheat grain, while LMW glutenin subunits (LMW-GSs) account for about 50% of gluten proteins and determine 30% of the technological quality [3]. With the separation of glutenin proteins on a polyacrylamide gel, it is possible to distinguish four regions of protein bands: A, B, C, and D [4]. The region A consists of HMW-GSs, while the LMW-GSs are located in regions B, C, and D. Additionally, in the C and D regions, there are α-, γ-, and ω-gliadins. The synthesis of proteins belonging to the LMW-GS group is mainly controlled by *Glu-3* loci located on the short arms of the first group of chromosomes, in the vicinity of the *Gli-1* loci complex responsible for coding of both γ- and ω-gliadins [3]. In recent years, based on the qualitative analysis, increased protein content in grains of wheat, which resulted in the presence of certain allelic variants encoding LMW-GSs, was observed [5]. In addition, biochemical and rheological analyses enabled the demonstration of both positive and negative effects of individual LMW subunit encoded by the *Glu-3* loci on the technological parameters of wheat flour, dough, and finished bread [5,6,7]. It was also shown that the presence of LMW-GSs encoded by the *Glu-A3* loci positively affects the viscoelastic properties, while the subunits encoded by the *Glu-B3* loci are important in the creation of mechanical parameters of dough (sodium dodecyl sulfate [SDS] sedimentation index, dough mixing time, dough resistance, and the ratio of dough work to resistance) [8,9]. So far, capillary zone electrophoresis (CZE) and reverse-phase high-performance liquid chromatography (RP-HPLC) methods were used to identify LMW-GSs in a very limited range. In previous studies, scientists only distinguished a group of proteins without detailed identification or identified individual LMW-GS [10,11,12,13,14].

The objective of this study was to identify HMW-GSs using sodium dodecyl sulfate-polyacrylamide gel electrophoresis (SDS-PAGE) and LMW-GSs in common wheat (*Triticum aestivum* L.) using CZE and RP-HPLC and to investigate their effect on the technological properties of wheat dough. The compositions of HMW-GSs and LMW-GSs were characterized in the analyzed plant material. Moreover, the technological quality of wheat and rheological analyses using a TA.XT Plus Texture Analyzer with Kieffer Rig (Stable MicroSystem) was determined at the microscale.

## 2 Materials and methods

### 2.1 Plant material

The plant material consisted of 57 varieties and 13 breeding lines of winter wheat (*Triticum aestivum* L.). The experimental material was cultivated at Smolice Plant Breeding Station in the years 2010–2012. In the first year, plant material was tested for the identification of HMW-GSs and LMW-GSs. In the last year, plant material was tested for technological quality. Plants were grown on 10 m^2^ in two replications, each on podzolic soil with clayey soil class IIIa. Peas were used as forecrop, and the fertilization dose was, respectively, 110 N, 60 P, and 90 K (kg ha^−1^) each year. Standard plant protection products against fungal diseases and pests were used during the experiment. The material was harvested to avoid inaccuracies.

Additionally, 22 reference varieties were used in this study (Table 1). This material was obtained from the Australia Winter Cereals Collection and is recommended as a standard for LMW-GSs [15]. These varieties were homozygous, with a strictly defined composition of LMW-GSs.

**Table 1:** List of the 22 foreign reference varieties with a strictly defined composition of LMW-GSs

Varieties	Alleles			Country of origin
	*Glu-A3*	*Glu-B3*	*Glu-D3*	
Alva	*a*	*d*	*a*	Portugal
Arcane	*c*	*c*	*a*	France
Bastian	*a*	*i*	*a*	France
Chara	*b*	*b*	*b*	Australia
Cheyenne	*c*	*e*	*f*	USA
Chinese Spring	*a*	*a*	*a*	China
Democrat	*a*	*h*	*a*	France
Gabo	*b*	*b*	*b*	Australia
Gluclub	*e*	*d*	*a*	Australia
Insygnia	*f*	*c*	*c*	Australia
Isis	*e*	*f*	*a*	Australia
Jufy-1	*e*	*i*	*d*	Belgium
Kharkov	*e*	*g*	*a*	Russia
Kukri	*d*	*h*	*b*	Australia
Newbury	*c*	*c*	*c*	UK
Norin-61	*d*	*i*	*c*	Japan
Norstar	*c*	*b*	*b*	Canada
Orca	*d*	*d*	*e*	France
Pato Argentino	*d*	*i*	*e*	Argentina
Radja	*e*	*f*	*b*	France
Rescue	*f*	*h*	*a*	Canada
Thatcher	*e*	*h*	*e*	Canada

### 2.2 Extraction of HMW glutenins and SDS-PAGE separation

The characterization of HMW-GSs was performed according to the methods of Tohver [16]. Material for protein extraction was wheat flour obtained from milling a single grain. HMW-GSs were extracted according to the procedure described by Salmanowicz [17] and Dai et al. [18]. The supernatants were transferred to clean microcentrifuge tubes (1.5 mL) and stored at 4°C until separation. A total of 7 µL of the supernatants were loaded onto stacking gel, including 4.5% (w/v) acrylamide, and HMW-GS proteins were separated on resolving gel containing 11.5% (w/v) acrylamide. SDS-PAGE was carried out using Protean II xi gel apparatus (Bio-Rad, Hercules, CA, USA) at 240 V for 4.5 h. Gels were stained overnight with Coomassie Brilliant Blue G-250.

### 2.3 Extraction of LMW glutenins and separation

LMW-GS proteins were extracted and analyzed using CZE and RP-HPLC techniques as three replicates. Material for protein extraction was obtained from flour that was obtained from milling a single grain. The LMW-GS fraction for the CZE analyses was extracted according to the method described by Salmanowicz et al. [19]. To carry out analyses with RP-HPLC, the LMW-GSs were extracted according to Salmanowicz [20] with some modifications [21]. Capillary electrophoretic separations of LMW-GSs were carried out on the P/ACE apparatus with the Beckman Coulter MDQ system. Silica capillaries with an internal diameter of 50 μm and a total length of 30.2 cm were used for the separation of proteins (the detector length was 21 cm). A Beckman Coulter absorbance UV detector was used for detection. For camera operation, parameter control, and initial analysis of results, the computer software GOLD System version 8.11 (Beckman Coulter) was used. The separation was run at a constant temperature of 38°C and 10 kV. The duration of the separation was 18 min. Detection of proteins occurred at a wavelength of 200 nm, in accordance with Di Luccia et al. [10]. Before each injection, the capillary was washed with 0.1 N hydrochloric acid (0.3 MPa for 4 min) and water (0.3 MPa for 1 min). The buffers and solutions used for the analyses were filtered through membranes and then sonicated. A buffer consisting of 20% acetonitrile (AcN), 0.2% polyvinylpyrrolidone (PVP-360), 0.05% hydroxypropyl methylcellulose, 0.05 M iminodiacetic acid, and lauryl sulfobetaine (SB-12) was used to fill the capillary. For the partition buffer, a mixture of 20% AcN, 0.15% poly(ethylene oxide), 0.05% IDA, and 26 mM SB-12 was used. Testing was carried out at the anode end of the capillary, for 3 s at 0.5 psi (3.447 × 10^−3^ MPa).

The chromatographic separation was carried out according to Li Vigni et al. [22] with some modifications [13]. Chromatographic separations of proteins isolated from glutenin extracts were carried out using a Beckman Coulter RP-HPLC apparatus equipped with two pumps (126 solvent module) and a UV spectral detector. For the separation of LMW-GSs, a chromatographic Phenomenex 250 C18 column (size 4.6 × 250 mm) was used. All solvents and reagents were filtered through a 0.5 µm Millipore (Bedford, MA, USA) membrane filter and sonicated before each analysis. A gradient of two solvents was used to separate the proteins on the chromatographic column: solvent (A) – ultrapure water with TFA (trifluoroacetic acid) (99.9/0.1%, v/v) and solvent (B) – ultrapure AcN by the addition of TFA (99.9/0.1%, v/v). Extracts were separated with increasing concentration of solvent B from 20 to 60% for 50 min and then to 80% for 5 min. Each time, prior to the analysis, the chromatography column was purged for 3 min under an increased flow of 80% AcN. Camera operation, parameter control, and initial analysis of the results were carried out using the GOLD Nouveau Chromatography Workstation version 1.7 software (Beckman Coulter).

### 2.4 Preparing the flour for rheological analysis

At the first step of the experiment, an initial assessment and determination of the basic physicochemical parameters of wheat grain were made using the standard near-infrared (NIR) technique [23]. The percentages of protein (%) and moisture (%) were determined for each wheat grain simple. About 300 g of grain samples was adjusted to 14% moisture content with water and stored for 72 h at 18^o^C prior to milling with a quad-roller mill (Quadrumat Junior). A drum sieve with a mesh size of 250 μm was used to separate the flour from the bran. The milling capacity was 300 g in 4 min, while the maximum extract was about 70%. The obtained flour was packed in paper bags, sealed, and stored for 14 days at 18^o^C. After that time, the percentages of protein and moisture of wheat flour were made using the standard NIR technique.

### 2.5 Rheological analysis

At the later step of the experiment, analyses were performed at the microscale using a TA.XT Plus Texture Analyzer. About 10 g flour and 2% brine were added into the mixer chamber. The required volume of brine for each sample was calculated by the Remix 32 program, by the following equation: water absorption (%, 14%mb) = protein (14%mb) × 1.5 × 43.6, based on previously determined protein content. The dough was prepared by mixing for 10 min. The dough obtained was formed into balls, covered tightly with foil, and rested in a heat chamber at 30°C for 30 min. After that time, the dough ball was placed in a mold with five grooves (53 mm × 5 mm × 3 mm). The mold was placed in a clamp, squeezed, excess dough was removed, and kept in a heat chamber for another 10 min. After that time, the formed dough strips were analyzed in extension at a crosshead speed of 3.3 mm s^−1^ and a trigger force of 5 g [24]. Parameters obtained from the Kieffer force–distance curves were maximum resistance (*R*_max_, in grams), maximum extensibility (*L*_max_, in millimeters), and area under the force versus distance curve (*P*_max_, in grams × millimeters). Each sample was analyzed in triplicate, of which the average was calculated. Due to the small sample amounts available, reference varieties were not used in the rheological analyses.


**Ethical approval:** The conducted research is not related to either human or animal use.

## 3 Results

### 3.1 Identification of HMW-GS

**Figure 1:**
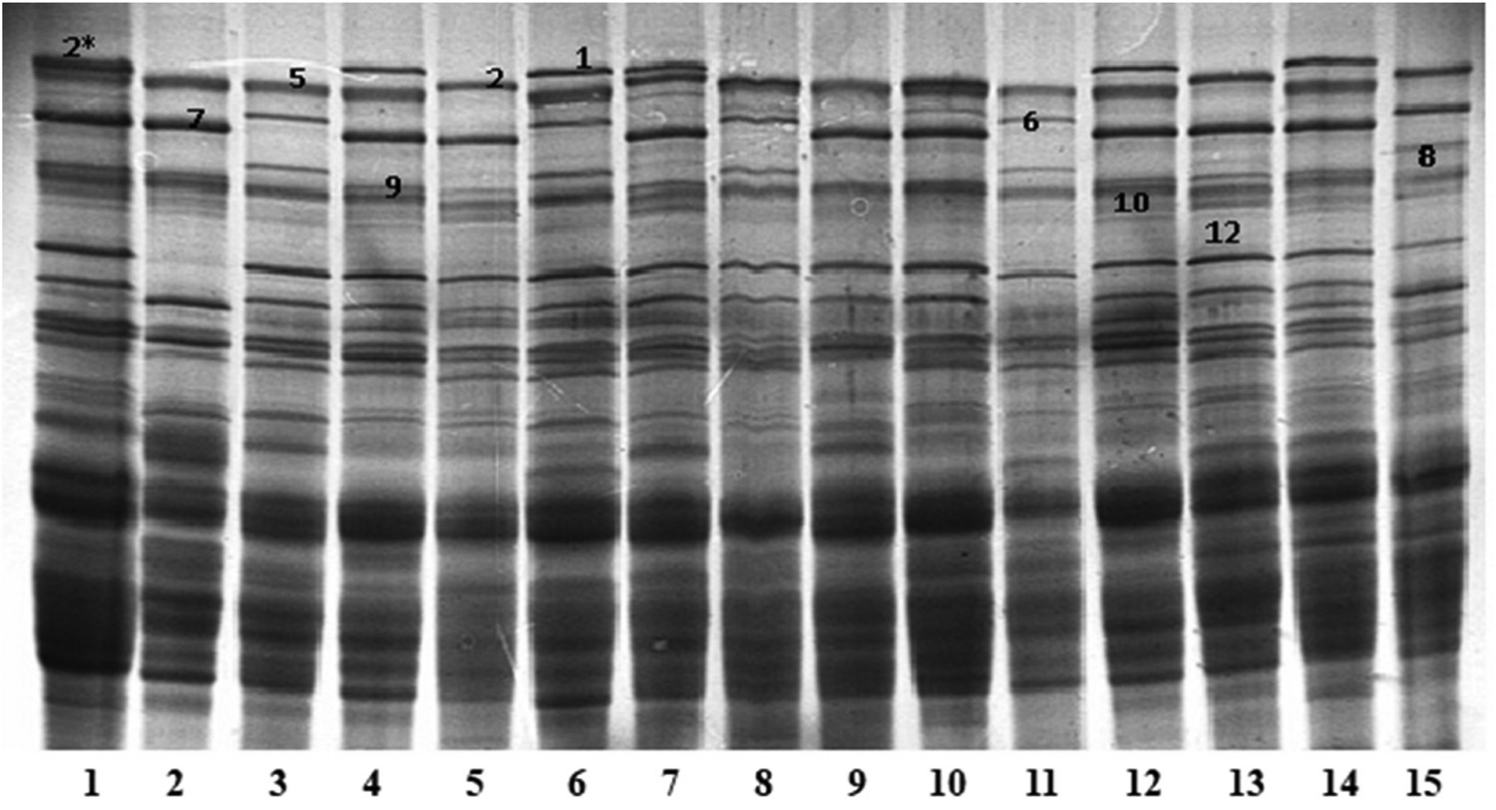
Electrophoretic images of dissociated HMW wheat glutenin subunits separated by SDS-PAGE. Lines: 1. Tonacja (2*/7+9/2+12), 2. Bamberka (N/7+9/5+10), 3. Ludwig (N/6+8/5+10), 4. Figura (1/7+9/5+10), 5. BZ 210801 (N/7+9/2+12), 6. Look (1/6+8/5+10), 7. Opus (1/7+9/2+12), 8. Batuta (N/6+8/5+10), 9. SZD 96 (N/7+9/5+10), 10. SMH 8063 (2*/7+9/5+10), 11. Discus (N/6+8/5+10), 12. Akteur (1/7+9/5+10), 13. Skagen (N/7+9/2+12), 14. KWS Ozon (1/7+9/5+10), 15. Bagou (N/6+8/2+12).

The use of the SDS-PAGE method allowed the identification of 10 HMW subunits that occurred in the plant material. Based on the analyses of the obtained images separated by electrophoresis of the subunits, eight HMW-GS schemas were distinguished. Figure 1 presents examples of electrophoretic images obtained for selected wheat. The electrophoretic mobility of individual subunits was referenced to the reference variety Tonacja containing Ax2*/Bx7+By9/Dx2+Dy12 subunits. HMW-GS patterns identified in the studied plant material are listed in Table 2. The HMW-GS scheme N/7+9/5+10 was the most frequent, occurring in 19 samples, constituting 27.14% of the analyzed plant material. Schemes 2*/7+9/5+10 and 1/6+8/5+10 were, jointly, the least common, with each occurring in only three samples (4.29%).

**Table 2:** Percentage of HMW-GS patterns in 70 tested varieties and lines of wheat

HMW-GS scheme			Number of varieties	Percentage
*Glu-A1*	*Glu-B1*	*Glu-D1*		
N	7+9	5+10	19	27.14
N	7+9	2+12	11	15.71
1	7+9	5+10	9	12.86
1	7+9	2+12	4	5.71
2*	7+9	5+10	3	4.29
N	6+8	5+10	11	15.71
N	6+8	2+12	10	14.29
1	6+8	5+10	3	4.29

### 3.2 Identification of LMW-GSs

Reference varieties of wheat representing the LMW subunit encoding alleles were used to determine the migration times for 52 major protein peaks via CZE analyses. The migration times of all protein peaks encoded by the *Glu-A3*, *Glu-B3,* and *Glu-D3* loci that were identified in the reference material are listed in Table 3. These results served to identify the LMW-GS alleles in the tested varieties.

**Table 3:** Migration time of protein peaks juxtaposed in blocks for the *Glu-3* alleles determined based on CZE profiles of wheat reference varieties

Genome A		Genome B				Genome D	
Allele	Migration time (min)	Allele	Migration time (min)	Allele	Migration time (min)	Allele	Migration time (min)
*Glu-A3a*	9.10	*Glu-B3a*	10.50	*Glu-B3e*	9.70	*Glu-D3a*	9.71
	12.14		10.70		10.45		13.38
			11.45		11.15	*Glu-D3b*	9.61
			11.78		11.60		13.57
*Glu-A3b*	9.44	*Glu-B3b*	10.47	*Glu-B3f*	10.41	*Glu-D3c*	9.64
	14.79		10.73		10.64		13.39
			11.39		11.36		
*Glu-A3c*	14.53	*Glu-B3c*	10.22	*Glu-B3g*	10.28	*Glu-D3d*	9.40
			10.49		11.23		13.50
			10.69		12.13		
			11.31	*Glu-B3h*	10.35	*Glu-D3e*	9.70
*Glu-A3d*	10.16	*Glu-B3d*	9.35		10.69		13.43
	13.24		10.38		11.40		
	14.85		11.33	*Glu-B3i*	9.93	*Glu-D3f*	9.85
*Glu-A3e*	—		11.65		10.37		13.87
*Glu-A3f*	12.83				11.33		

**Figure 2:**
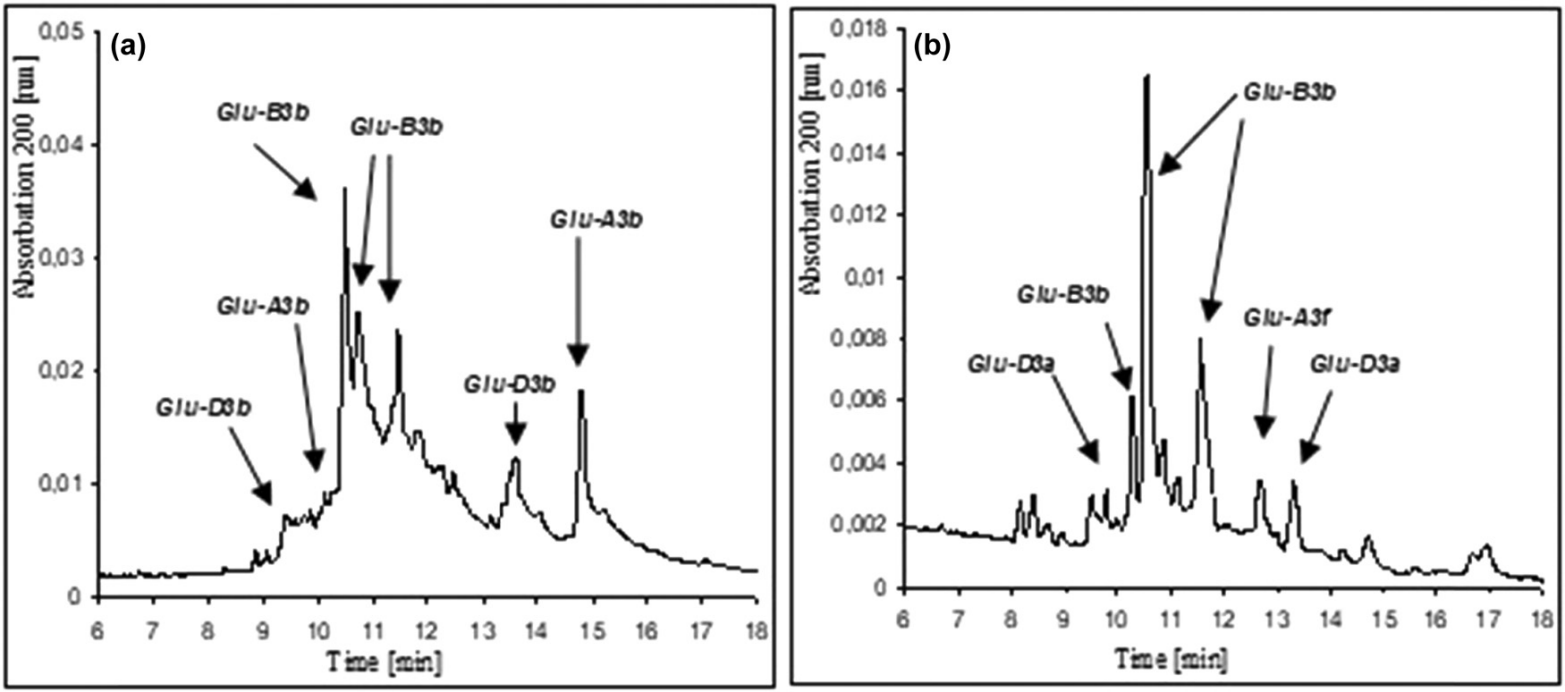
Identification of LMW-GSs using capillary electrophoresis in (a) Gabo and (b) Jantarka.

Figure 2 presents the identification of LMW-GS samples using capillary electrophoresis in reference wheat Gabo (*Glu-A3b*, *Glu-B3b*, *Glu-D3b*) and previously uncharacterized wheat variety Jantarka (*Glu-A3f*, *Glu-B3b*, *Glu-D3a*).

Sixteen allelic combinations of the *Glu-3* loci, based on separation visualized on CZE electropherograms, were distinguished in the 70 tested kinds of wheat. The exact distribution of plant material according to HMW-GSs and LMW-GSs is presented in Appendix Table 1 (Table A1). Four alleles were detected for the *Glu-A3* and *Glu-D3* loci (*Glu-A3b*, *Glu-A3d*, *Glu-A3e*, *Glu-A3f* and *Glu-D3a*, *Glu-D3b*, *Glu-D3c*, *Glu-D3e*, respectively), whereas for the *Glu-B3* locus, seven alleles were identified (*Glu-B3a*, *Glu-B3b*, *Glu-B3c*, *Glu-B3d*, *Glu-B3e*, *Glu-B3h*, and *Glu-B3i*). Across the studied plant material, the most common allele was *Glu-D3c* present in 52 samples, followed by *Glu-A3e* and *Glu-A3f* which were found in 33 samples, and *Glu-B3b* present in 27 samples. Infrequent variants included *Glu-B3i* present only in Bogatka, SZD 96, and SZD 205, and *Glu-B3a* was found in Banderola, Brilliant, Look, and Turkis; while the *Glu-A3d* allele was found only in Bystra, and the *Glu-D3b* allele only in line SZD 205.

Verification of the identified LMW-GS schemes in the studied plant material was achieved by chromatographic analyses. First, 22 reference varieties were scored against the LMW-GS fraction (Table 1). As a result, for the *Glu-A3* locus (*Glu-A3a-d* and *Glu-A3f*), three to five protein peaks were observed on the chromatograms of the reference samples, which elute from 39.53 to 44.97 min. No peaks were observed for the *Glu-A3e* allele due to the lack of expression of this allele. For the reference varieties containing the *Glu-B3h* allele, four peaks were observed that eluted from 41.22 to 48.92 min. For the *Glu-B3* locus (*Glu-3a – Glu-B3g* and *Glu-B3i*), five protein peaks were observed on the chromatograms, which eluted from 40.69 to 48.92 min. Sets of LMW-GS protein peaks encoded by the *Glu-D3* locus alleles consisted of three peaks with retention times ranging from 40.51 to 50.46 min. The elution times of all protein peaks encoded by the *Glu-A3*, *Glu-B3*, and *Glu-D3* loci that were identified in the reference material are listed in Table 4.

**Table 4:** Elution time of protein peaks juxtaposed in blocks for the *Glu-3* alleles determined based on RP-HPLC profiles of wheat reference varieties

Genome A		Genome B				Genome D	
Allele	Elution time (min)	Allele	Elution time (min)	Allele	Elution time (min)	Allele	Elution time (min)
*Glu-A3a*	40.55	*Glu-B3a*	41.57	*Glu-B3e*	41.77	*Glu-D3a*	43.07
	41.56		42.60		42.15		46.55
			45.05		42.67		50.33
						*Glu-D3b*	42.85
*Glu-A3b*	39.53		48.27		45.00		46.64
	43.28		48.92		48.05		50.27
		*Glu-B3b*	40.96	*Glu-B3f*	40.69	*Glu-D3c*	43.20
			41.82		41.63		46.69
			42.35		42.63		50.29
			45.08		45.15		
*Glu-A3c*	40.69		48.33		48.24		
		*Glu-B3c*	41.72	*Glu-B3g*	41.37	*Glu-D3d*	41.92
			42.57		41.95		46.62
*Glu-A3d*	39.89		43.73		42.55		50.32
	40.41		45.14		45.18		
	43.16		48.22		48.05		
				*Glu-B3h*	41.22	*Glu-D3e*	42.81
		*Glu-B3d*	40.88		42.27		46.58
					45.22		50.19
*Glu-A3e*	—		41.20		48.15		
			41.63	*Glu-B3i*	41.44	*Glu-D3f*	40.51
			45.20		42.27		46.75
*Glu-A3f*	44.97		48.70		43.55		50.46
					45.08		
					48.09		

The use of RP-HPLC made it possible to identify the same amount of LMW-GSs, both in the reference material and in the studied plant material, as in the case of applied CE. As was the case for capillary electrophoresis, alleles of the *Glu-3* loci constituting 16 allelic variants were found in the examined material.

### 3.3 Rheological analysis of wheat dough

**Figure 3:**
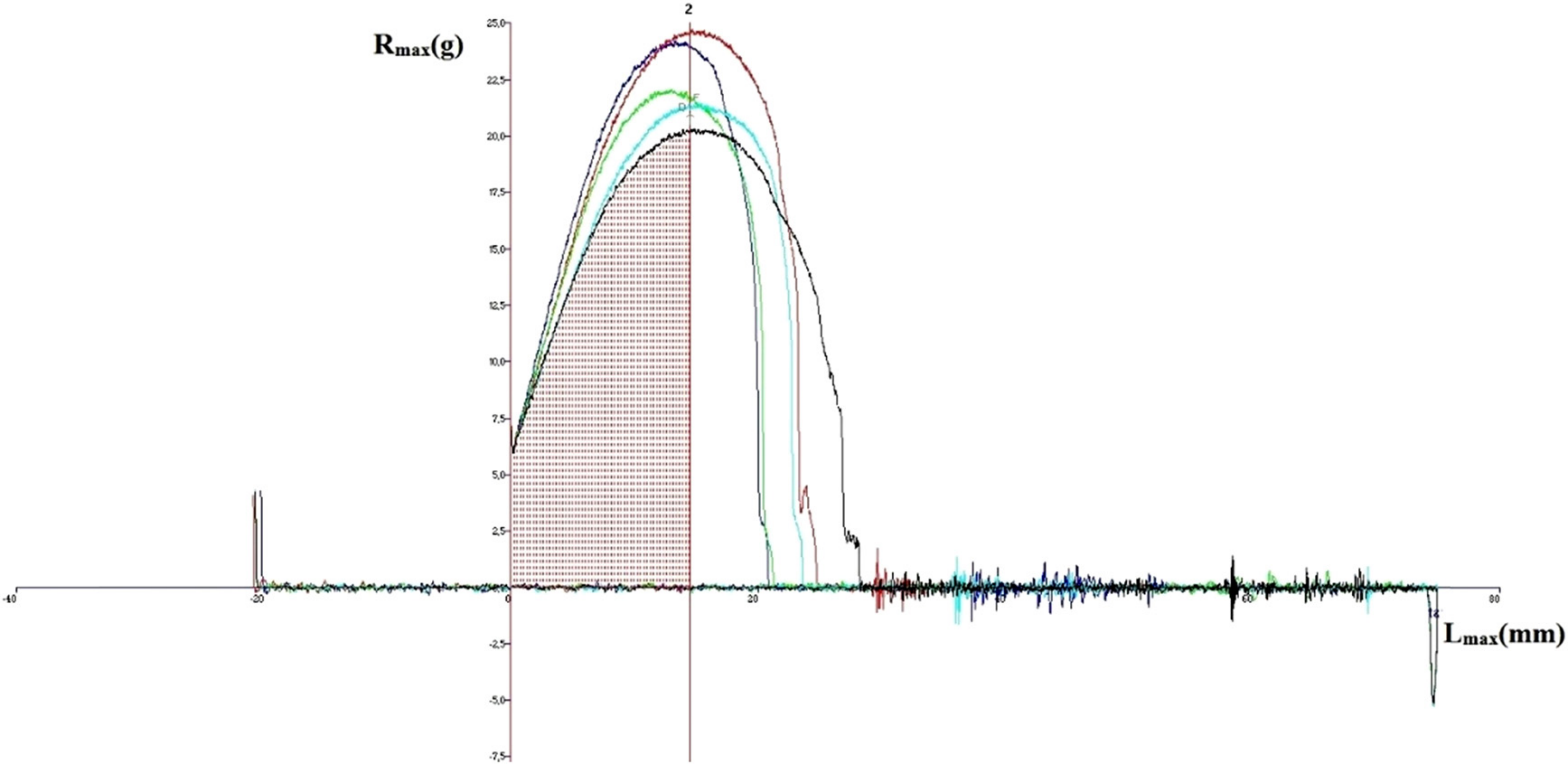
Graph obtained on the texture analyzer for variant containing *Glu-A3f*, *Glu-B3b*, and *Glu-D3e* (*R*_max_ – resistance, *L*_max_ – extension).

The average values of dough parameters determined by the Kieffer method for allelic variations of the *Glu-3* loci are presented in Table 6, and an example of a graph obtained using the Kieffer texture analyzer is shown in Figure 3.

The results obtained were subjected to an analysis of variance to determine the effect of the LMW-GS variant on the rheological features of the dough (Table 5).

**Table 5:** Analysis of variance for the qualitative determinants of the dough of the tested wheat varieties and breeding lines

Parameter	Sources of variation	D. F.	Mean square	*F* statistics	*F* 0.05	*F* 0.01
*R*_max_	Variant LMW	15	0.3313	2.747	1.72	2.13
*L*_max_	Variant LMW	15	0.7014	3.091	1.72	2.13
*P*_max_	Variant LMW	15	0.1308	3.765	1.72	2.13

Analyzing the tested samples in terms of average resistance values (*R*_max_), significant differences were found between the allelic variants of LMW-GSs (*P* < 0.01). With an average dough resistance value of *R*_max_ = 25.89 g for the first group (number^1^ in Table 6) (variants: *Glu-A3f*, *Glu-B3b*, and *Glu-D3e*; *Glu-A3f*, *Glu-B3e*, and *Glu-D3e*; *Glu-A3f*, *Glu-B3b*, and *Glu-D3a*; *Glu-A3f*, *Glu-B3c*, and *Glu-D3c*; and *Glu-A3e*, *Glu-B3h*, and *Glu-D3e*) and an average resistance of *R*_max_ = 34.71 g for the second group (number^2^ in Table 6). Similarly, two groups of allelic combinations were discriminated with respect to the mean values of elongation (*L*_max_). The first group (number^3^ in Table 6) with an average extension of 17.89 mm was wheat doughs characterized by the alleles: *Glu-A3f, Glu-B3b,* and *Glu-D3e*; *Glu-A3d, Glu-B3b,* and *Glu-D3c; Glu-A3e, Glu-B3a,* and *Glu-D3c*; *Glu-A3f, Glu-B3c,* and *Glu-D3c*; *Glu-A3f, Glu-B3b,* and *Glu-D3a; Glu-A3e, Glu-B3d,* and *Glu-D3c; Glu-A3e, Glu-B3i,* and *Glu-D3c;* and *Glu-A3e, Glu-B3b,* and *Glu-D3c*. The second group (number^4^ in Table 6) with an average extension of 21.71 mm was the remaining eight variants of the *Glu-3* loci. In the case of surface area, *P*_max_ significant differences were found between the allelic variants of LMW-GSs (*P* < 0.01). Two groups were distinguished, the first (number^5^ in Table 6) with *P*_max_ = 335,86 g mm and variants: *Glu-A3f*, *Glu-B3b, and Glu-D3e; Glu-A3f, Glu-B3b,* and *Glu-D3a; Glu-A3f, Glu-B3c,* and *Glu-D3c; Glu-A3d*, *Glu-B3b,* and *Glu-D3c; Glu-A3e, Glu-B3a,* and *Glu-D3c*; and the second (number^6^ in Table 6) with an average *P*_max_ = 505.92 g mm for the 11 remaining analyzed variants.

**Table 6:** Average values of rheological parameters determined by the Kieffer method for allelic variations of the *Glu-3* loci

*Glu-A3*	*Glu-B3*	*Glu-D3*	*R*_max_ (g)	*L*_max_ (mm)	*P*_max_ (g mm)
*b*	*h*	*c*	35.45^2^	22.40^4^	544.73^6^
*d*	*b*	*c*	34.86^2^	16.90^3^	389.59^5^
*e*	*a*	*c*	33.14^2^	17.32^3^	401.47^5^
*e*	*b*	*c*	32.68^2^	19.35^3^	462.94^6^
*e*	*d*	*c*	34.31^2^	18.83^3^	449.25^6^
*e*	*h*	*c*	36.97^2^	21.95^4^	583.98^6^
*e*	*h*	*e*	29.29^1^	22.15^4^	463.71^6^
*e*	*i*	*a*	38.39^2^	20.73^4^	536.35^6^
*e*	*i*	*b*	36.20^2^	23.03^4^	578.98^6^
*e*	*i*	*c*	35.14^2^	18.85^3^	443.26^6^
*f*	*b*	*a*	27.60^1^	17.70^3^	348.42^5^
*f*	*b*	*e*	18.65^1^	14.56^3^	221.98^5^
*f*	*c*	*c*	28.65^1^	17.33^3^	361.33^5^
*f*	*d*	*c*	36.78^2^	20.95^4^	532.96^6^
*f*	*e*	*c*	35.39^2^	21.22^4^	533.42^6^
*f*	*e*	*e*	25.09^1^	24.19^4^	450.76^6^

Note: In the table, 1–6 are groups of allelic combinations of LMW-GS which were discriminated with respect to mean values of resistance (*R*_max_), elongation (*L*_max_) and surface area (*P*_max_).

The smallest total mean resistance (*R*_max_ = 18.65 g) came from a wheat dough with the allelic variant *Glu-A3f*, *Glu-B3b*, and *Glu-D3e*. In turn, the highest total average resistance, and thus the greatest resistance (*R*_max_ = 38.39 g), was in a dough with an allelic variant of *Glu-A3e*, *Glu-B3i*, and *Glu-D3a*. The weakest dough in terms of extensibility (*L*_max_ = 14.56 mm) was observed for allelic variants: *Glu-A3f*, *Glu-B3b*, and *Glu-D3e*. The most flexible and stretchable (*L*_max_ = 24.19 mm) was from dough with the allele variant *Glu-A3f*, *Glu-B3e*, and *Glu-D3e*. Taking into account the area of *P*_max_, the smallest cumulative mean values (221.98 g mm) were for the *Glu-A3f*, *Glu-B3b*, and *Glu-D3e* variants, while the largest total mean values (583.98 g mm) were wheat doughs characterized by the allele variant *Glu-A3e*, *Glu-B3a*, and *Glu-D3c*.

## 4 Discussion

Literature reports show that the technological properties of wheat are largely determined by the composition and the amount of gluten, which includes glutenins and gliadins. A thorough understanding of the polymorphism of glutenin proteins makes it possible to determine the extent to which the rheological properties of wheat dough are determined by a complex of gluten proteins formed by both high- and low-molecular subunits [25,26]. HMW-GSs account for up to 10% of gluten proteins, and it has been shown that they determine up to 70% of the quality characteristics of wheat grain. Otherwise, the impact of LMW-GSs on rheological parameters is still poorly characterized. It seems that the specific rheological properties of wheat dough can also be significantly affected by LMW-GSs, which account for up to 50% of gluten and generate up to 30% variation in technological features of wheat [1,4].

Protein electrophoresis in polyacrylamide gel with the addition of sodium dodecyl sulfate (SDS-PAGE) is a commonly used method for assessing the variability of the qualitative composition of wheat storage proteins. The comparative analyses of protein profiles obtained on SDS-electropherograms allowed us to distinguish 11 HMW-GSs in the examined plant material, coded by the allelic variants of the genes in the *Glu-1* locus: *Glu-A1-1a* (Ax1), *Glu-A1-1b* (Ax2*), *Glu-A1-1c* (Null variant), *Glu-B1-1a* (Bx7), *Glu-B1-1d* (Bx6), *Glu-B1-2a* (By8), *Glu-B1-2d* (By9), *Glu-D1-1a* (Dx5), *Glu-D1-2a* (Dx2), *Glu-D1-1d* (Dy5), and *Glu-D1-2b* (Dy12). Featured HMW subunits are usually identified in wheat technological studies [27,28].

In the presented work, the identification of the LMW-GS qualitative composition in the tested material was carried out using electrophoretic (CZE) and chromatographic (RP-HPLC) methods. In recent years, CZE has been used to perform qualitative and quantitative determinations of the majority of distinguished classes of wheat storage proteins [17,19,29]. The literature data show that LMW-GSs migrate in the silica capillaries in the time range similar to the HMW-GS fraction, which requires prior accurate separation of these fractions prior to conducting the separation [30]. Li et al. [11], based on identified protein peaks, distinguished two alleles at the *Glu-A3* locus, four alleles at the *Glu-B3* locus, and three alleles at the *Glu-D3* locus. In the presented study, a wide range of migration times (9.10–14.85 min) was found for individual LMW subunits, which enabled their full identification in the tested genotypes. In contrast to the studies of Li et al. [11], we observed one to four protein peaks corresponding to LMW-GSs based on the CZE electropherograms with the exception of the *Glu-A3e* allele, which was not expressed. Multiple migration times of the individual LMW-GSs result from the presence of multiple genes at a particular locus [11].

At the same time, the RP-HPLC method was used to determine the qualitative composition of LMW-GSs in tested wheat varieties with the previously determined HMW-GS composition. LMW subunits have so far been characterized using this method by several research teams, but the subject of research has been a very small number of trials in individual studies [12,31]. For the separation of LMW subunits, researchers applied various fillings in the chromatographic columns, which makes it more difficult to compare the retention times of protein peaks on the presented chromatograms. The main disadvantage of this method when compared with free capillary electrophoresis is a long time of separation of individual samples (up to 60 min) and high costs of columns and solvents used for protein separation. The LMW subunit separation carried out as part of this study, refining the methodology and using the most modern columns, enabled full identification of all (from one to five) subunits encoded by particular *Glu-3* loci. The reports presented previously revealed the presence of only a few subunits but confirm that multiple elution times of LMW-GS proteins are due to the presence of multiple genes at a particular locus [12,31]. In our study, full concordance was obtained in the number of subunits in separation performed using the CZE and RP-HPLC methods. In recent years, along with the refinement of the RP-HPLC method, i.e., the use of ultra-dry liquid chromatography (UPLC), the use of columns with smaller fillings has provided a comparable resolution of proteins for a number of chemical compounds with three times shorter subunit separation time. Yu et al. [13] using the UPLC method to separate the LMW subunits shortened the time of separation (up to 18 min) but did not obtain such a good resolution for individual subunits (only 1–2 subunits were distinguished) as in the case of the methodology used in the presented study.

To determine the performance traits of common wheat, technological research studies on grain or flour are carried out [32,33,34,35]. In recent years, the impact of LMW-GSs on rheological parameters has mainly been determined using a texture analyzer with the Kieffer method [36]. The rheological analyses carried out in this research confirmed the increase in dough resistance in the LMW-GS containing samples encoded by the *Glu-A3d, Glu-B3d, Glu-B3h, Glu-B3i, Glu-D3a,* and *Glu-D3b* alleles. The lowest dough resistance was linked with the presence of *Glu-A3e* and *Glu-A3f* alleles. The increase in dough resistance in wheat genotypes containing LMW-GSs encoded by the *Glu-B3i* allele has not previously been reported in the literature. Based on previous studies by the teams of Branlard et al. [37] and Eagles et al. [38], it can be concluded that the increased extensibility and elongation of dough also depends on the presence of LMW subunits coded at the *Glu-A3* locus (*Glu-A3a, Glu-A3d*), the *Glu-B3* locus (*Glu-B3b* and *Glu-B3d*), and the *Glu-D3* locus (*Glu-D3b, Glu-D3c*). Rai et al. [32] showed that LMW-GSs coded at the *Glu-A3* locus (*Glu-A3b, Glu-A3c*) and the *Glu-B3* (*Glu-B3b*) locus are responsible for the low rheological values. In subsequent studies, the beneficial effects of *Glu-A3d, Glu-B3d, Glu-B3b, Glu-B3f* alleles, and *Glu-D3c* [39] on the elongation of dough were demonstrated. Maucher et al. [40] found that the improvement of the dough elongation parameter is influenced by the presence of the *Glu-A3b, Glu-B3d, Glu-D3d* allelic combination. Oury et al. [41] have shown that LMW-GSs encoded by the *Glu-A3a, Glu-B3g, Glu-D3a,* and *Glu-D3b* alleles increase the extensibility of the dough. Park’s team [42] demonstrated the positive effects of the *Glu-A3b, Glu-A3d, Glu-B3b, Glu-B3d, GluD3b,* and *Glu-D3a* alleles on the dough extensibility. The rheological data obtained in this study indicated that particularly beneficial effects on the extensionality of the dough came from the *Glu-B3b, Glu-D3b, Glu-D3c* alleles, which confirms the results obtained by other researchers [38,39,41]. The presence of the *Glu-A3b, Glu-A3f, Glu-B3h,* and *Glu-B3e* alleles in samples also had a significant influence on the shaping of this parameter. In-depth studies on the area under the force versus distance curve (*P*_max_) were conducted by Maucher’s team [40]. They arranged the LMW-GS coding alleles favorably affecting this parameter in the following order: *Glu-A3d > Glu-A3c > Glu-A3b > Glu-A3e* at the *Glu-A3* locus; *Glu-B3d > Glu-B3g > Glu-B3h > Glu-B3f > Glu-B3i* at the *Glu-B3* locus and *Glu-D3d > Glu-D3b > Glu-D3a > Glu-D3c* at the *Glu-D3* locus. Oury et al. [41] observed an increase in tear resistance in LMW-GS-tested samples encoded by the *Glu-A3d*, *Glu-B3b’, Glu-B3g* and *Glu-D3b* alleles. In a recent study, Zhang’s team [43] ranked the *Glu-3* loci alleles contribution to the increase in tear dough resistance within the *Glu-A3* locus as *Glu-A3c > Glu-A3d > Glu-A3f > Glu-A3b > Glu-A3e;* within the *Glu-B3* locus as *Glu-B3i > Glu-B3b = Glu-B3a > Glu-B3f = Glu-B3g > Glu-B3h > Glu-B3c > Glu-B3d*; and within the *Glu-D3* locus as *GluD3a = Glu-D3b = Glu-D3c > Glu-D3d > Glu-D3f*. Based on comparative analyses carried out in this work, it was shown that the presence of *Glu-A3f, Glu-A3e, Glu-B3a, Glu-B3e, Glu-B3h, Glu-D3a, Glu-D3b,* and *Glu-D3c* alleles in the tested samples has a significant effect on increasing the breaking strength of the dough. The data obtained on the beneficial effects of LMW-GSs encoded by the *Glu-B3h*, *Glu-D3a* and *Glu-D3b* alleles on breaking strength are in agreement with the literature data [40,43]. In the case of the remaining alleles of Glu-3 loci in the tested samples, the presence of which significantly enhanced the tear strength of the dough, discrepancies were found with the literature data, which may be due to the smaller number of samples tested by other authors.

## 5 Conclusion

The use of modern analytical methods such as capillary electrophoresis and RP-HPLC enabled the full identification of LMW-GSs encoded by the *Glu-3* loci alleles. Our research clearly showed that LMW-GSs play an important role in creating the rheological quality of wheat. Obtained results enabled the selection of wheat varieties containing the *Glu-3* loci scheme (*Glu-A3b, Glu-A3f* at the *Glu-A3* locus; *Glu-B3a, Glu-B3b, Glu-B3d, Glu-B3h* at the *Glu-B3* locus; *Glu-D3a, Glu-D3c* at the *Glu-D3* locus) determining the most beneficial quality parameters, namely, Operetka, Smaragd, SMH 90, Ludwig, Brilliant, Natula, Akteur, and Bamberka. These varieties may be used in wheat breeding for crossing and developing new plants with favorable technological parameters. This research can be integrated with a molecular marker approach in an expansion of the knowledge about the genetic background of wheat quality giving an effective marker-assisted selection in the future.

## Appendix

**Table A1:** List of wheat genotypes according to HMW-GS and LMW-GS schemes

Sample number	Genotypes	HMW-GS scheme	LMW-GS scheme		
			*Glu-A3*	*Glu-B3*	*Glu-D3*
1	AREZZO	N/7+9/5+10	*f*	*e*	*c*
2	BALETKA		*e*	*h*	*c*
3	BAMBERKA		*e*	*d*	*c*
4	BOCKRIS		*e*	*b*	*c*
5	BOGATKA		*e*	*i*	*c*
6	BRILLIANT		*e*	*a*	*c*
7	CUBUS		*e*	*b*	*c*
8	DOROTA		*f*	*d*	*c*
9	KOHELIA		*e*	*h*	*e*
10	KRANICH		*f*	*e*	*c*
11	PAMIER		*f*	*c*	*c*
12	SKAGEN		*f*	*e*	*c*
13	SMARAGD		*f*	*d*	*c*
14	SZD 205		*e*	*i*	*b*
15	SZD 87		*e*	*d*	*c*
16	SZD 96		*e*	*i*	*a*
17	TORAS		*f*	*e*	*c*
18	TORRILD		*f*	*e*	*c*
19	TURKIS		*e*	*a*	*c*
20	ANTHUS	N/7+9/2+12	*f*	*e*	*e*
21	BB 742206 DH		*e*	*b*	*c*
22	BZ 210801		*f*	*c*	*c*
23	FIDELIUS		*e*	*b*	*c*
24	GARANTUS		*e*	*d*	*c*
25	KREDO		*e*	*b*	*c*
26	POB 779 05		*e*	*h*	*e*
27	RUMBA		*e*	*h*	*c*
28	RYSA		*e*	*h*	*c*
29	SMH 8134		*e*	*h*	*c*
30	VISCOUNT		*e*	*b*	*c*
31	ATTLAS	N/6+8/5+10	*f*	*b*	*a*
32	BUTEO		*f*	*c*	*c*
33	BYSTRA		*d*	*b*	*c*
34	DISCUS		*f*	*b*	*e*
35	GALVANO		*b*	*h*	*c*
36	GECKO		*f*	*b*	*a*
37	JANTARKA		*f*	*b*	*a*
38	LP 227 1 03		*f*	*b*	*a*
39	LUDWIG		*b*	*h*	*c*
40	PREMIO		*f*	*c*	*c*
41	ACONEL	N/6+8/2+12	*e*	*b*	*c*
42	ADONIS		*f*	*e*	*c*
43	AND 3509		*e*	*b*	*c*
44	AUGUSTUS		*f*	*e*	*c*
45	BAGOU		*f*	*b*	*e*
46	BISCAY		*f*	*b*	*e*
47	CENTENAIR		*e*	*b*	*c*
48	HENRIK		*f*	*b*	*a*
49	KATART		*f*	*b*	*e*
50	MULAN		*e*	*d*	*c*
51	AKTEUR	1/7+9/5+10	*e*	*h*	*c*
52	ARISTOS		*e*	*b*	*c*
53	BANDEROLA		*e*	*a*	*c*
54	FIGURA		*f*	*e*	*c*
55	KWS OZON		*e*	*b*	*c*
56	NATULA		*e*	*h*	*c*
57	QUEBON		*f*	*e*	*c*
58	SMH 92		*e*	*b*	*c*
59	STETANUS		*e*	*b*	*c*
60	KEPLER	1/7+9/2+12	*f*	*b*	*e*
61	OPERETKA		*f*	*d*	*c*
62	OPUS		*f*	*b*	*a*
63	TUAREG		*f*	*b*	*a*
64	LOOK	1/6+8/5+10	*e*	*a*	*c*
65	POTENTIAL		*f*	*e*	*c*
66	SZD 11		*e*	*d*	*c*
67	SMH 8063	2*/7+9/5+10	*f*	*c*	*c*
68	SMH 90		*b*	*h*	*c*
69	SMUGA	2*/7+9/2+12	*f*	*c*	*c*
70	GLOBAL	2*/6+8/5+10	*f*	*b*	*a*

## References

[1] Rasheed A, Xia X, Yan Y, Appels R, Mahmood T, He Z. Wheat seed storage proteins: Advances in molecular genetics, diversity and breeding applications. J Cereal Sci. 2014;60(1):11–24. 10.1016/J.JCS.2014.01.020.

[2] Podolska G, Aleksandrowicz E, Szafrańska A. Bread making potential of Triticum aestivum and Triticum spelta species. Open Life Sci. 2020;15(1):30–40. 10.1515/biol-2020-0004.

[3] D’Ovidio R, Masci S. The low-molecular-weight glutenin subunits of wheat gluten. J Cereal Sci. 2004;39(3):321–39. 10.1016/j.jcs.2003.12.002.

[4] Gale KR. Diagnostic DNA markers for quality traits in wheat. J Cereal Sci. 2005;41(2):181–92. 10.1016/J.JCS.2004.09.002.

[5] Li Y, Zhou R, Branlard G, Jia J. Development of introgression lines with 18 alleles of glutenin subunits and evaluation of the effects of various alleles on quality related traits in wheat (Triticum aestivum L.). J Cereal Sci. 2010;51(1):127–33. 10.1016/j.jcs.2009.10.008.

[6] Jin H, Zhang Y, Li G, Mu P, Fan Z, Xia X, et al. Effects of allelic variation of HMW-GS and LMW-GS on mixograph properties and Chinese noodle and steamed bread qualities in a set of Aroona near-isogenic wheat lines. J Cereal Sci. 2013;57(1):146–52. 10.1016/J.JCS.2012.10.011.

[7] Liang D, Tang J, Peña RJ, Singh R, He X, Shen X, et al. Characterization of CIMMYT bread wheats for high- and low-molecular weight glutenin subunits and other quality-related genes with SDS-PAGE, RP-HPLC and molecular markers. Euphytica. 2010;172(2):235–50. 10.1007/s10681-009-0054-x.

[8] Barak S, Mudgil D, Khatkar BS. Relationship of gliadin and glutenin proteins with dough rheology, flour pasting and bread making performance of wheat varieties. LWT Food Sci Technol. 2013;51(1):211–7. 10.1016/j.lwt.2012.09.011.

[9] Hernández ZJE, Figueroa JDC, Rayas-Duarte P, Martínez-Flores HE, Arámbula GV, Luna GB, et al. Influence of high and low molecular weight glutenins on stress relaxation of wheat kernels and the relation to sedimentation and rheological properties. J Cereal Sci. 2012;55(3):344–50. 10.1016/J.JCS.2012.01.009.

[10] Di Luccia A, Lamacchia C, Mamone G, Picariello G, Trani A, Masi P, et al. Application of capillary electrophoresis to determine the technological properties of wheat flours by a glutenin index. J Food Sci. 2009;74(4):C307–11. 10.1111/j.1750-3841.2009.01117.x. 19490316

[11] Li J, Wang S, Yu Z, Li X, Guo G, Feng S, et al. Optimization and development of capillary electrophoresis for separating and identifying wheat low molecular weight glutenin subunits. J Cereal Sci. 2012;55:254–6. 10.1016/j.jcs.2011.12.005.

[12] Liu W, Zhang Y, Gao X, Wang K, Wang S, Zhang Y, et al. Comparative proteome analysis of glutenin synthesis and accumulation in developing grains between superior and poor quality bread wheat cultivars. J Sci Food Agric. 2012;92(1):106–15. 10.1002/jsfa.4548. 21815156

[13] Yu Z, Han C, Yan X, Li X, Jiang G, Yan Y. Rapid characterization of wheat low molecular weight glutenin subunits by ultraperformance liquid chromatography (UPLC). J Agric Food Chem. 2013;61(17):4026–34. 10.1021/jf400472s. 23560948

[14] Dangi P, Khatkar BS. Extraction and purification of low molecular weight glutenin subunits using size exclusion chromatography. J Food Sci Technol. 2019;56(2):951–6. 10.1007/s13197-018-03560-1. PMC6400785 30906052

[15] Gupta RB, Shepherd KW. Two-step one-dimensional SDS-PAGE analysis of LMW subunits of glutelin – 1. Variation and genetic control of the subunits in hexaploid wheats. Theor Appl Genet. 1990;80(1):65–74. 10.1007/BF00224017. 24220812

[16] Tohver M. High molecular weight (HMW) glutenin subunit composition of some Nordic and Middle European wheats. Genet Resour Crop Evolution. 2007;54(1):67–81. 10.1007/s10722-005-1885-5.

[17] Salmanowicz BP. Detection of high molecular weight glutenin subunits in triticale (×Triticosecale Wittm.) cultivars by capillary zone electrophoresis. J Agric Food Chem. 2008;56(20):9355–61. 10.1021/jf8016546. 18808142

[18] Dai S, Xu D, Yan Y, Wen Z, Zhang J, Chen H, et al. Characterization of high- and low-molecular-weight glutenin subunits from Chinese Xinjiang wheat landraces and historical varieties. J Food Sci Technol. 2020;57(10):3823–35. 10.1007/s13197-020-04414-5. PMC7447723 32904055

[19] Salmanowicz BP, Langner M, Mrugalsk B, Ratajczak D, Górny AG. Grain quality characteristics and dough rheological properties in Langdon durum-wild emmer wheat chromosome substitution lines under nitrogen and water deficits. J Sci Food Agric. 2016;97(7):2030–41. 10.1002/jsfa.8006. 27558295

[20] Salmanowicz BP. Primary structure and polymorphism of 2S albumins from seeds of Andean lupin (Lupinus mutabilis Sweet). Eur Food Res Technol. 1999;209(6):416–22. 10.1007/s002170050519.

[21] Langner M, Franaszek S, Salmanowicz B. Detection of LMW glutenin genes of the Glu-3 locus in some polish wheat cultivars by capillary electrophoresis and RP-HPLC. 10th Symposium on High-Performance Separation Methods. Siofok: Hungarian Society for Separation Sciences; 2015. p. 98.

[22] Li Vigni M, Baschieri C, Marchetti A, Cocchi M. RP-HPLC and chemometrics for wheat flour protein characterisation in an industrial bread-making process monitoring context. Food Chem. 2013;139(1–4):553–62. 10.1016/j.foodchem.2013.01.085. 23561145

[23] Demichelis M, Vanzetti LS, Crescente JM, Nisi MM, Pflüger L, Bainotti CT, et al. Significant effects in bread-making quality associated with the gene cluster glu-D3/Gli-D1 from the bread wheat cultivar prointa Guazú. Cereal Res Commun. 2019;47(1):111–22. 10.1556/0806.46.2018.055.

[24] Dobraszczyk BJ, Salmanowicz BP, Ługowska B, Chełkowski J. Rapid quality assessment of wheat cultivars registered in Poland using the 2-g mixograph and multivariate statistical analysis. Cereal Chem. 2005;82(2):182–6. 10.1094/CC-82-0182.

[25] León E, Marín S, Giménez MJ, Piston F, Rodríguez-Quijano M, Shewry PR, et al. Mixing properties and dough functionality of transgenic lines of a commercial wheat cultivar expressing the 1Ax1, 1Dx5 and 1Dy10 HMW glutenin subunit genes. J Cereal Sci. 2009;49(1):148–56. 10.1016/J.JCS.2008.08.002.

[26] Yang FP, Wang LH, Wang JW, He XY, Zhang XK, Shang XW, et al. Characterisation of high- and low-molecular-weight glutenin subunit genes in Chinese winter wheat cultivars and advanced lines using allele-specific markers and SDS-PAGE. Crop Pasture Sci. 2009;61(1):84–91. 10.1071/CP09164.

[27] Gianibelli MC, Gupta RB, Lafiandra D, Margiotta B, MacRitchie F. Polymorphism of high Mr glutenin subunits in triticum tauschii: characterisation by chromatography and electrophoretic methods. J Cereal Sci. 2001;33(1):39–52. 10.1006/jcrs.2000.0328.

[28] Shewry PR, Popineau Y, Lafiandra D, Belton P. Wheat glutenin subunits and dough elasticity: findings of the EUROWHEAT project. Trends Food Sci Technol. 2000;11(12):433–41. 10.1016/S0924-2244(01)00035-8.

[29] Salmanowicz BP, Langner M, Franaszek S. Charge-based characterisation of high-molecular-weight glutenin subunits from common wheat by capillary isoelectric focusing. Talanta. 2014;129:9–14. 10.1016/j.talanta.2014.04.055. 25127558

[30] Herrero M, García-Cañas V, Simo C, Cifuentes A. Recent advances in the application of capillary electromigration methods for food analysis and foodomics. Electrophoresis. 2010;31(1):205–28. 10.1002/elps.200900365. 19967713

[31] Peña E, Bernardo A, Soler C, Jouve N. Relationship between common wheat (Triticum aestivum L.) gluten proteins and dough rheological properties: gluten proteins and rheological properties in wheat. Euphytica. 2005;143(1–2):169–77. 10.1007/s10681-005-3157-z.

[32] Rai A, Singh AM, Ganjewala D, Kumar RR, Ahlawat AK, Singh SK, et al. Rheological evaluations and molecular marker analysis of cultivated bread wheat varieties of India. J Food Sci Technol. 2019;56(4):1696–707. 10.1007/s13197-019-03593-0. PMC6443729 30996405

[33] Dangi P, Chaudhary N, Khatkar BS. Rheological and microstructural characteristics of low molecular weight glutenin subunits of commercial wheats. Food Chem. 2019;297:124989. 10.1016/j.foodchem.2019.124989. 31253302

[34] Bonilla JC, Erturk MY, Kokini JL. Understanding the role of gluten subunits (LMW, HMW glutenins and gliadin) in the networking behavior of a weak soft wheat dough and a strong semolina wheat flour dough and the relationship with linear and non-linear rheology. Food Hydrocoll. 2020;108:106002. 10.1016/j.foodhyd.2020.106002.

[35] Du X, Wei J, Luo X, Liu Z, Qian Y, Zhu B, et al. Low-molecular-weight glutenin subunit LMW-N13 improves dough quality of transgenic wheat. Food Chem. 2020;327:127048. 10.1016/j.foodchem.2020.127048. 32454285

[36] Langner M, Krystkowiak K, Salmanowicz BP, Adamski T, Krajewski P, Kaczmarek Z, et al. The influence of Glu-1 and Glu-3 loci on dough rheology and bread-making properties in wheat (Triticum aestivum L.) doubled haploid lines. J Sci Food Agric. 2017;97(15):5083–91. 10.1002/jsfa.8385. 28429474

[37] Branlard G, Dardevet M, Saccomano R, Lagoutte F, Gourdon J. Genetic diversity of wheat storage proteins and bread wheat quality. Euphytica. 2001;119(1–2):59–67. 10.1023/A:1017586220359.

[38] Eagles HA, Eastwood RF, Hollamby GJ, Martin EM, Cornish GB. Revision of the estimates of glutenin gene effects at the Glu-B1 locus from southern Australian wheat breeding programs. Austr J Agric Res. 2004;55(10):1093–6. 10.1071/AR04113.

[39] Ma W, Appels R, Bekes F, Larroque O, Morell MK, Gale KR. Genetic characterisation of dough rheological properties in a wheat doubled haploid population: additive genetic effects and epistatic interactions. Theor Appl Genet. 2005;111(3):410–22. 10.1007/s00122-005-2001-0. 15965651

[40] Maucher T, Figueroa JDC, Reule W, Peņa RJ. Influence of low molecular weight glutenins on viscoelastic properties of intact wheat kernels and their relation to functional properties of wheat dough. Cereal Chem. 2009;86(4):372–5. 10.1094/CCHEM-86-4-0372.

[41] Oury FX, Chiron H, Faye A, Gardet O, Giraud A, Heumez E, et al. The prediction of bread wheat quality: Joint use of the phenotypic information brought by technological tests and the genetic information brought by HMW and LMW glutenin subunits. Euphytica. 2009;171(1):87–109. 10.1007/s10681-009-9997-1.

[42] Park CS, Kang CS, Jeung JU, Woo SH. Influence of allelic variations in glutenin on the quality of pan bread and white salted noodles made from Korean wheat cultivars. Euphytica. 2011;180(2):235–50. 10.1007/s10681-011-0385-2.

[43] Zhang X, Jin H, Zhang Y, Liu D, Li G, Xia X, et al. Composition and functional analysis of low-molecular-weight glutenin alleles with Aroona near-isogenic lines of bread wheat. BMC Plant Biol. 2012;12:243. 10.1186/1471-2229-12-243. PMC3562532 23259617

